# Microbially produced vitamin B12 contributes to the lipid-lowering effect of silymarin

**DOI:** 10.1038/s41467-023-36079-x

**Published:** 2023-01-30

**Authors:** Wen-Long Sun, Sha Hua, Xin-Yu Li, Liang Shen, Hao Wu, Hong-Fang Ji

**Affiliations:** 1 Institute of Biomedical Research, School of Life Sciences and Medicine, Shandong University of Technology, Zibo, Shandong China; 2 Department of Cardiology, Ruijin Hospital/Luwan Branch, Shanghai Jiao Tong University School of Medicine, South Chongqing Rd. No. 149, Shanghai, China; 3 State Key Laboratory of Genetic Engineering, Human Phenome Institute, and Department of Bariatric and Metabolic Surgery, Huashan Hospital, Fudan University, Shanghai, China; 4 Zhangjiang Fudan International Innovation Center, Fudan University, Shanghai, China; 5 School of Life Sciences, Ludong University, Yantai, Shandong China

**Keywords:** Microbiome, Non-alcoholic fatty liver disease, Pharmacology

## Abstract

Silymarin has been used for improving hepatic damage and lipid disorders, but its action mechanism remains to be clarified. Here, we investigate the contributions of the gut microbiota to the improvement of liver lipid metabolism by silymarin. We find i) strong and significant microbial shifts upon silymarin but not silibinin treatment; ii) over 60% variations of liver fat are explained by silymarin-induced bacterial B12 production in male rats but not in male germ-free mice; iii) fecal microbiota transplantation confirms their protective roles against liver fat accumulation; iv) upregulation of one-carbon metabolism and fatty acid degradation pathways are observed based on the liver transcriptome analyses; and v) in humans the delta changes of serum B12 associate negatively with the fluctuations of serum triglycerides. Overall, we reveal a mechanism of action underpinning the lipid-lowering effect of silymarin via the gut microbiota and its vitamin B12 producing capabilities.

## Abstract (web summary)

Silymarin has been used for improving hepatic damage and lipid disorders, but its action mechanism remains to be clarified. Here, the authors reveal a mechanism of action underpinning the lipid-lowering effect of silymarin via the gut microbiota and its vitamin B12 producing capabilities.

## Introduction

Silymarin, a mixture of flavonolignans (with silibinin being the major component^1^) and some other polyphenolic compounds such as taxifolin^2^, has long been known with diverse hepatoprotective properties such as antioxidant and lipid-lowering effects^3,4^ and could improve non-alcoholic fatty liver diseases (NAFLD)^5,6^. The corresponding clinical studies of silymarin have been comprehensively reviewed recently^7^. Although moderate improvements on liver functions and fibrosis have been observed, controversies exist among reported clinical trials potentially due to differences in sample size and treatment duration^7^. In addition, the dose effects and poor absorption observed for this drug might also play a role and it has been observed that the concentrations required for many of the known pharmacological effects of silymarin are hardly achievable in both animals (~0.73%)^8^ and humans (~1–2%)^9^. Thus, the action mechanisms of silymarin and its hepatoprotective benefits warrant further elucidation. Of interest, after oral administration, silymarin similar to metformin^10,11^, has been observed with a high concentration in the gastrointestinal tract. Whether the lipid-lowing effect of silymarin works through gut microbiota resembling the glucose-lowering effect of metformin^12^ still needs further investigation.

To address this, we studied the improvement of liver lipid metabolism after supplementation of silymarin or silibinin for 12 weeks in obese rats and profiled the gut microbiota on collected fecal samples using 16s rRNA sequencing. A subset of fecal DNA samples was then subjected to whole genomics shotgun (WGS) and targeted metabolomics for characterization of the microbial functional changes upon silymarin treatment. We also performed the germ-free mice and fecal microbiota transplantation (FMT) experiments to explore the causality and liver RNA sequencing to identify the potential target genes in the liver by the gut microbes and associated metabolites.

## Results

### Improved liver lipid metabolism by silymarin

**Fig. 1:**
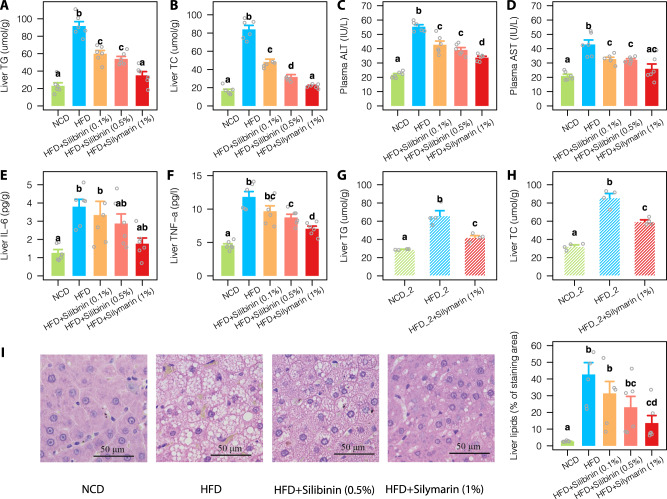
Improvement of liver lipid metabolism in rats upon silymarin/silibinin treatment for 12 weeks. Levels of liver TG (**A**) and TC (**B**); levels of plasma ALT (**C**) and AST (**D**); and levels of liver IL-6 (**E**) and TNF-α (**F**) in all five treatment groups. Changes of Liver TG (**G**) and TC (**H**) from a second replicate experiment. **I** Representative microphotograph of hematoxylin and eosin (H&E) staining and quantification of liver lipids. Values were shown as mean ± S.E.M. Groups labeled with different letters (a, b, c, or d) indicate significant statistical differences (FDR adjusted *P* < 0.05; two-sided Wilcox rank-sum test). Six rats were included in each group. Scale bar: 50 μm. NCD normal chow diet, HFD high fat diet, ALT alanine transaminase, AST aspartate aminotransferase, TG triglycerides, TC total cholesterol, IL-6 interleukin 6, TNF-α tumor necrosis factor alpha. Source data are provided as a Source data file.

In contrast with the control rats fed with high-fat diet (HFD; 45% calorie from fat mostly composed of lard), extra supplementation with silymarin (1%) could effectively prevent liver triglycerides (TG) and total cholesterol (TC) accumulation (Fig. 1A, B). However, there was no significant differences in body weight gain and food intake between the silymarin and HFD control groups (Supplementary Fig. 1A, B). The attenuated liver lipid accumulation upon silymarin treatment was paralleled by improved liver function as reflected from reduced levels of plasma alanine transaminase (ALT) and aspartate transaminase (AST) (Fig. 1C, D). The liver concentrations of interleukin 6 (IL-6) and tumor necrosis factor alpha (TNF-α), the two key inflammatory markers associated with NAFLD, were also reduced (Fig. 1E, F). As expected, silymarin treatment was also observed with improved plasma lipid profiles (Supplementary Fig. 1C, D). The two groups of rats fed with HFD and different doses of silibinin, the major active component of silymarin, demonstrated comparable improvement of lipid metabolism although to a lesser extent than that from silymarin treatment (Fig. 1A–F and Supplementary Fig. 1C, D). In addition, improvement of liver lipid metabolism by silymarin was confirmed in a second replicate experiment (Fig. 1G, H) and by histological analyses of the liver tissues (Fig. 1I).

### Altered gut microbial composition by silymarin

**Fig. 2:**
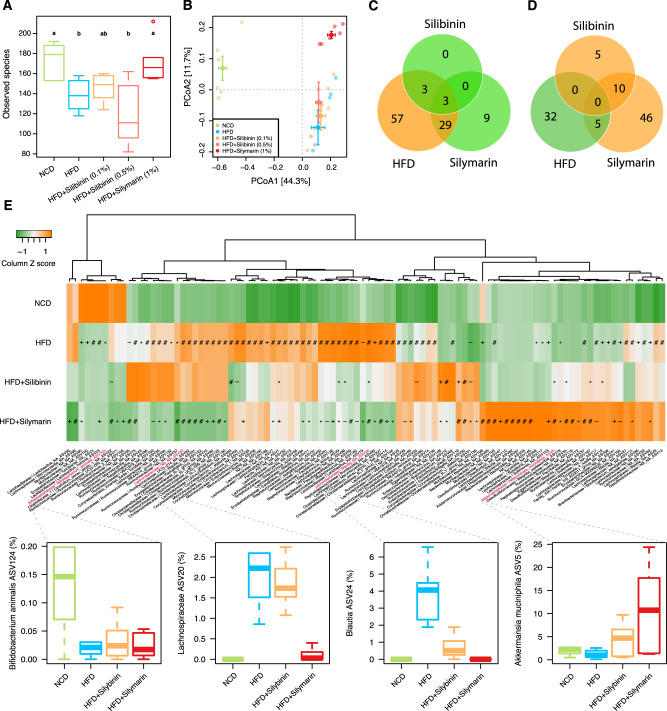
Microbial compositional changes in different treatment groups. Boxplots showing the alpha diversity measured by observed species (**A**) and the principal coordinate analysis (**B**) of the gut microbiota. **C**, **D** Venn plots showing the numbers of significantly increased (colored in yellow) and decreased (in green) amplicon sequence variations (ASVs) in HFD vs. NCD and silymarin/silibinin vs. HFD groups; the two silibinin groups were combined for this analysis. **E** Heatmap showing the mean relative abundances of the top 100 ASVs in different groups; changes in the relative abundances of four representative ASVs were further highlighted. Six rats were included in each group. Two-sided Wilcox rank-sum test was used to access the group differences of alpha diversity; groups labeled with different letters (a, b, or c) indicate significant statistical differences (FDR adjusted *P* < 0.05). Significant different ASVs were analyzed by Wald test (−, *, +, and # indicate FDR adjusted *P* < 0.1, 0.05, 0.01, and 0.001, respectively). The horizontal line in each box represents the median, the top and bottom of the box the 25th and 75th percentiles, and the whiskers 1.5 times the interquartile range. NCD normal chow diet, HFD high fat diet. Source data are provided as a Source data file.

To investigate whether the gut microbiota was also changed upon silymarin/silibinin treatment, we performed 16s rRNA gene (V3-V4 region) sequencing in all five groups of rats on collected fecal samples. Consistent with other studies^13–15^, obesogenic HFD resulted in reduced alpha diversity as assessed by the observed species (Fig. 2A). Silymarin but not silibinin treatment increased the richness of the bacterial species. However, principal coordinate analysis (PCoA) revealed that silymarin treatment shifted the gut microbiota toward a different direction rather than restoring it to a level resembling that in the rats fed with normal-chow diet (NCD) (Fig. 2B). In total, the relative abundance of 92 amplicon sequence variations (ASVs, species with 100% 16s similarity) were found significantly increased in HFD vs. NCD groups; of which, 35 (38%) were significantly reduced upon silymarin/silibinin treatment but most (29 ASVs) were regulated by silymarin only (Fig. 2C). In contrast, only 37 ASVs were observed with reduced abundance in HFD vs. NCD groups and only 5 (13.5%) of them could be restored with silymarin treatment; interestingly, the abundance of 56 ASVs were found largely driven by silymarin treatment, including 10 (66.7%) out of 15 that were found increased in silibinin vs. HFD groups (Fig. 2D).

Hierarchical clustering of the top 100 abundant ASVs that were significantly altered across groups demonstrated four distinct patterns of microbial compositional changes (Fig. 2E; Supplementary Data 1): (1) bacteria whose abundance were reduced by HFD but couldn’t be restored by silymarin/silibinin such as *Bifidobacterium animalis* (ASV124); (2) bacteria that were increased by HFD but can only be rescued by silymarin not silibinin (e.g., ASV20, which belongs to Lachnospiraceae); (3) those that can be rescued by both silymarin and silibinin (e.g., ASV24 annotated as *Blautia*); (4) bacteria whose changes were largely driven by silymarin and/or silibinin (e.g., ASV5 annotated as *Akkermansia muciniphila*). Intriguingly, of those five ASVs that belong to *Christensenellaceae R-7* group, only 1 showed increased abundance along improved lipid metabolism upon silymarin/silibinin treatment whereas the abundances of the other four were actually increased by HFD (Fig. 2E). *Christensenellaceae* had been associated with a lean phenotype in both rodents and humans^16^. However, our study here together with a few others^17^ showed some bacteria within this family might have positive connections with diet-induced obesity. It thus will be interesting to investigate in future whether this bacterial family contains distinct species/strains that can affect the host ‘obese’ phenotype in opposite directions. Taken together, our findings suggest that although silymarin can partly alleviate the HFD-induced gut microbial dysbiosis, other compounds within silymarin such as taxofolin or components resulted from microbial metabolism of silymarin rather than silibinin should play a major role in shaping the gut microbial composition. In support, most components but not silibinin within silymarin has been shown recently to be able to be substantially and rapidly metabolized by human gut microbiota^2^ and taxofolin also bears the potential to regulate the gut microbiota^18^.

### Silymarin-altered microbiota improves liver lipid metabolism

**Fig. 3:**
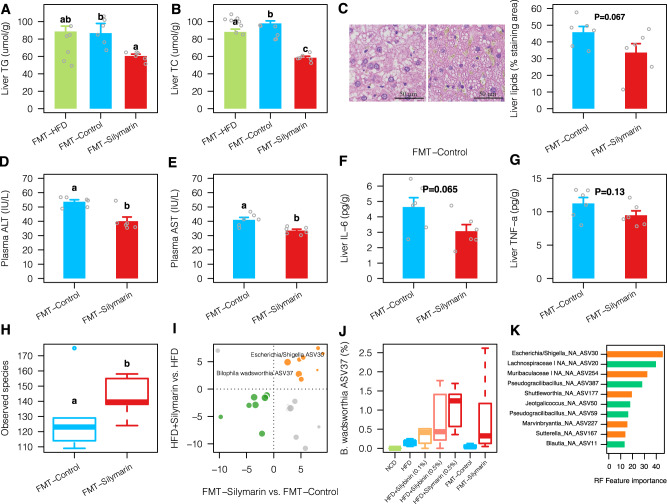
Liver lipids and associated gut microbiota changes after fecal microbiota transplantation (FMT) to rats fed on high-fat diet for 12 weeks. Levels of liver TG (**A**) and TC (**B**) together with representative microphotograph of hematoxylin and eosin (H&E) staining (**C**); levels of plasma ALT (**D**) and AST (**E**); and levels of liver IL-6 (**F**) and TNF-α (**G**) after FMT for 12 weeks using fecal samples collected from silymarin-treated rats with (FMT-control) or without heat-killing (FMT-Silymarin). Two-sided Wilcox rank-sum test was used to access the group differences; groups labeled with different letters (a, b, or c) indicate significant statistical differences (FDR adjusted *P* < 0.05). For bar plots, values were shown as mean ± S.E.M. **H** The differences of alpha diversity in FMT-Control vs. FMT-Silymarin (one outlier within the FMT-Control group as shown in the boxplot was removed for the statistical analysis). **I** The log2 fold changes of amplicon sequence variations (ASVs) that were significantly changed in both HFD + Silymatrin vs. HFD and FMT-Silymarin vs. FMT-control groups; the dot sizes were proportional to their prevalence among samples; dots colored in yellow and green indicate consistently increased and decreased ASVs, respectively. **J** Changes in the relative abundance of *B. wadsworthia* in all treatment groups. **K** The top 10 ASVs that contribute to most to the variations of liver TG as assessed by random forest regression based on all groups of rats (orange and green colors indicate increased and decreased abundance upon silymarin treatment, respectively). Six rats were included in each group. Scale bar: 50 μm. ALT alanine transaminase, AST aspartate aminotransferase, TG triglycerides, TC total cholesterol, IL-6 interleukin 6, TNF-α tumor necrosis factor alpha. Source data are provided as a Source data file.

To test whether there was a causal link between the silymarin-altered microbiota and improved liver lipid metabolism, we performed daily gavage of the fecal samples from silymarin group to two groups of HFD-fed rats. The first group of rats were transferred with heat-killed fecal microbiota; this group together with rats transferred with fecal microbiota from the HFD group served as the double control groups. After 12 weeks of FMT, the FMT-silymarin group was observed with significantly lower levels of liver TG and TC, as confirmed by histological analyses of the liver tissues (Fig. 3A–C). However, the mean liver TG difference between groups attributed to FMT was 28.1 μmol/g which was only half of that from direct silymarin treatment (56.6 μmol/g; Fig. 1A). Lower levels of plasma ALT and AST were also observed in the FMT-silymarin group (Fig. 3D, E). The inflammation marker IL-6 only showed a trend of difference, and no difference was found for TNF-α between groups (Fig. 3F, G), potentially, due to less pronounced improvement of liver lipid metabolism by FMT. Consistent with our results in the silymarin treatment experiment (Fig. 2A), the FMT-silymarin group was also observed with higher alpha diversity (Fig. 3H). 17 ASVs were found consistently changed between FMT-silymarin vs. FMT-control and HFD + silymarin vs. HFD (Fig. 3I). The two most prevalent ASVs that showed positive correlations with silymarin treatment were ASV30 affiliated to *Escherichia/Shigella* and *Bilophila wadsworthia* (ASV37). In agreement with previous findings^19,20^, we showed that *B. wadsworthia* was tightly connected with HFD but, to our surprise, its abundance was further increased to a greater extent by silymarin/silibinin treatment (Fig. 3J). Additional random forest regression analysis based on the 16 S rRNA profiles obtained from all groups of rats revealed that the ASV30 contributed most to the variation of liver TG (Fig. 3K). Further heat stability measurement of silymarin based on the high-performance liquid chromatography (HPLC) revealed that components of silymarin were quite stable under the given heating condition mentioned above (Supplementary Fig. 2), excluding a potential confounding effect from heat-induced silymarin metabolism. Our results here thus support the idea that silymarin-altered microbiota can directly contribute to the improved liver lipid metabolism.

### Improved liver lipid metabolism is coupled with enhanced bacterial B12 biosynthesis

**Fig. 4:**
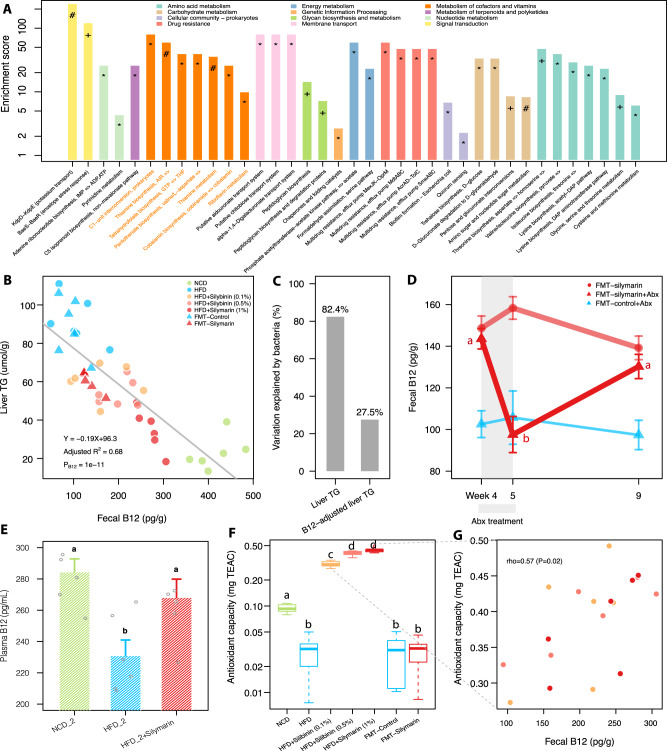
Microbial functional alternations upon silymarin treatment. **A** Pathway enrichment analysis for those 110 KOs that were mostly (spearman correlation coefficient rho ≥0.9 or ≤−0.9) associated with liver TG; hypergeometric test was used for analysis (*, +, and # indicate adjusted *P* < 0.05, 0.01, and 0.001, respectively). **B** Linear regression analysis between fecal B12 concentrations and liver TG levels. **C** Bar plot showing the variations of raw liver TG and liver TG adjusted for fecal B12 explained by the gut bacteria based on random forest regression; the levels of liver TG adjusted for B12 were measured by the residuals calculated from the linear regression between liver TG and fecal B12 in panel (**B**). **D** The longitudinal changes of fecal B12 during fecal microbiota transplantation (FMT) with or without antibiotic (Abx) treatment during weeks 4 to 5; the gut microbes transferred in the FMT-Control group were heat-killed. **E** Levels of plasma B12 in the replication experiment. **F** The fecal antioxidant capacities as measured by trolox equivalent antioxidant capacity (TEAC) assay in different treatment groups. **G** The correlation between the fecal B12 and antioxidant capacities in three groups of mice on HFD. Six rats were included in each group. Two-sided Wilcox rank-sum test was used in panels (**D**–**F**) to obtain the significance levels; groups labeled with different letters (a, b, or c) indicate significant statistical differences. For bar plots, values were shown as mean ± S.E.M. NCD normal chow diet, HFD high fat diet, TG triglycerides. Source data are provided as a Source data file.

To identify which bacterial gene pathways might account for the improvement of liver lipid metabolism, three fecal DNA samples from the HFD group and three from the HFD + silymarin group were subjected to WGS sequencing. 110 KEGG orthology groups (KOs) were found to be strongly associated with liver TG (spearman correlation coefficient rho ≥0.9 or ≤−0.9; *P* < 0.05) (Supplementary Data 2) and were significantly enriched for 37 different KEGG pathways (adjusted *P* < 0.05) (Fig. 4A); of these, 14 pathways were associated with ‘metabolism of cofactors and vitamins’ (especially B-group vitamins such as thiamine/B1, riboflavin/B2, folate/B9, and cobalamin/B12) and ‘amino acid metabolism’. We therefore performed a targeted measurement of those four types of B-group vitamins together with vitamins A and D3 on collected fecal samples. The concentrations of fecal vitamins A, B12, and D3 were found significantly changed upon silymarin/silibinin treatment (Supplementary Fig. 3). However, only fecal B12 significantly associated with liver TG base on linear regression analysis (Supplementary Fig. 4A) and explained 68% variations of the liver TG by itself (Fig. 4B). Of note, the increase of fecal B12 by FMT was much less than that by direct silymarin treatment, consistent with its less pronounced improvement of liver lipid metabolism (Fig. 3A, B). According to our random forest model (Figs. 3K and 4C), the bacteria could explain as high as 82.4% variations of the liver TG but the R^2^ value decreased to 27.5% when adjusting for contributions from fecal B12, confirming a major contribution from microbially produced B12 than other bacterial metabolites. In contrast, the alpha diversity, a marker reflecting host serum metabolites richness^21^, only explained 15.5% of the variation (Supplementary Fig. 4B) and was not significant after adjusting for fecal B12 (*P* = 0.29), suggesting that the silymarin-altered microbiota might improve liver lipid metabolism largely through bacterial B12 biosynthesis.

To further clarify whether the fecal B12 was mainly from bacterial biosynthesis or the diet, we performed a longitudinal monitoring of the fecal B12 in FMT-silymarin vs. FMT-control rats (Fig. 4D). Our results indicated that antibiotics treatment for one week can only reduce the fecal B12 in the FMT-silymarin but not FMT-control groups; importantly, the reduced level of fecal B12 upon antibiotics treatment in the FMT-silymarin group can be reversed by further FMT for another four weeks. In addition, the amount of B12 in our HFD (0.01 mg/kg diet) was only about 1/3 of that in the NCD (0.03 mg/kg), providing further evidence implying that the increased fecal B12 concentrations upon silymarin treatment most likely were derived from bacterial biosynthesis. We also showed that B12 synthesized in the gut can be absorbed into the plasma (Fig. 4E) and thus utilized by the host.

To address whether silymarin-adapted gut microbiota was enriched for B12 producing bacteria, 55 query sequences representing more than 40 key genes involved in bacterial B12 biosynthesis^22^ were blasted against 44.8 million proteins from over 12,000 bacteria with complete genomes (NCBI reference release 92) (Supplementary Data 3). Our results indicated that gut microbes with increased relative abundances upon silymarin treatment such as *Escherichia*/*Shigella* (ASV30), *Bilophila wadsworthia* (ASV37), *Akkermansia muciniphila* (ASV5), and *Flavonifractor plautii* (ASV119) indeed have more genes thus higher B12 producing capabilities than those decreased by silymarin including *Bifidobacterium animalis* (ASV124), ASVs from *Christensenellaceae* (e.g., ASV124), and ASVs belonging to *Lactobacillus* (e.g., ASV23 and 36) (Fig. 2E, Supplementary Data 1 and 3).

Since silymarin/silibinin mainly serves as antioxidant^23^ and bacterial B12 biosynthesis is very tightly regulated by the environmental redox potentials^24–26^, we hypothesized that the gut redox changes upon silymarin/silibinin treatment might partially explain the increased bacterial B12 production. Antioxidant capacity measurement revealed that HFD was associated with lower antioxidant capacity in the fecal samples whereas silymarin/silibinin treatment could greatly reduce the fecal redox potential (Fig. 4F) and correlated with fecal B12 concentrations (Fig. 4G; rho = 0.57, *P* = 0.02). However, no significant difference in terms of the fecal antioxidant capacities was observed in FMT-silymarin vs. FMT-control groups (Fig. 4F). This might address in part why the increase of fecal B12 and in return the improvement of liver lipid metabolism by FMT (Fig. 3A, B and Supplementary Fig. 3D) were not as dramatic as that by silymarin treatment (Fig. 1A, B and Supplementary Fig. 3D).

### B12 supplementation improves liver TG

**Fig. 5:**
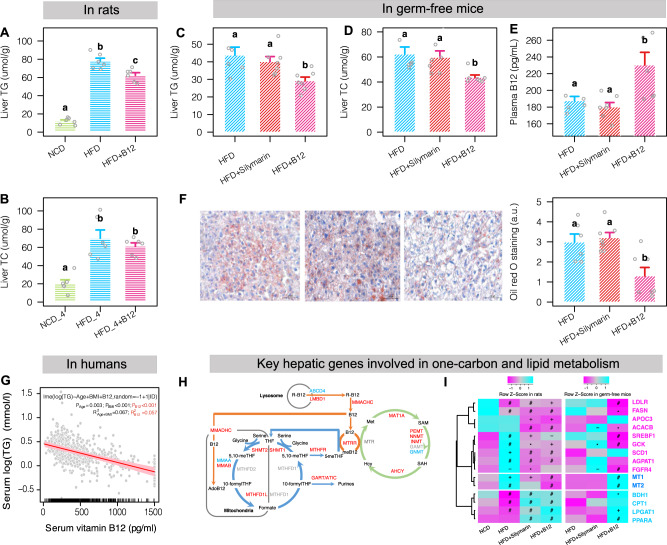
Levels of liver lipids in rats and germ-free mice supplemented with or without B12 and association of plasma TG and B12 in humans. Levels of liver TG (**A**) and TC (**B**) in rats supplemented with or without B12 in the diet (*n* = 6 for each group). Two-sided Wilcox rank-sum test; groups labeled with different letters (a, b, or c) indicate significant statistical differences. Levels of liver TG (**C**) and TC (**D**) and plasma B12 (**E**) in germ-free mice (*n* = 7, 8, and 7 for the HFD, HFD + silymarin, and HFD + B12 treated groups, respectively). **F** Oil Red O staining of liver lipid droplets in germ-free mice (*n* = 6, 7, and 6 for the HFD, HFD + silymarin, and HFD + B12 treated groups, respectively; FDR adjusted *P* value = 0.032 and 0.080 for HFD + B12 versus HFD + Silymarin and HFD groups, respectively). **G** Association between the levels of serum vitamin B12 and triglycerides in 398 responders using linear mixed effect model. **H** Rat liver transcriptome analyses revealing the upregulation of key genes involved the one-carbon metabolism mediated by folate, B12, and the methionine cycles. **I** Regulation of key hepatic genes involved in lipogenesis and fatty acid oxidation in rats and in germ-free mice by silymarin or B12 (−, *, +, and # indicate *P* values smaller than 0.1, 0.05, 0.01, and 0.001, respectively). Wald test implanted in DESeq2 package was used for identification of differentially expressed genes and hypergeometric test was used for pathway enrichment analysis. For bar plots, values were shown as mean ± S.E.M. Scale bar: 50 μm. NCD normal chow diet, HFD high fat diet, TG, triglycerides, TC total cholesterol. Source data are provided as a Source data file.

To investigate whether B12 plays direct roles in improving liver lipid metabolism, we then performed the B12 supplementation experiment. As shown in Fig. 5A, B, extra B12 supplementation (3 μM/kg body weight per day, calculated based on fecal B12 levels from silymarin treatment) for 12 weeks could indeed prevent liver TG but not TC accumulation. However, the B12-induced effects on liver lipid metabolism were less pronounced compared with those from direct silymarin administration (Fig. 1A, B). To examine whether this is due to inadequate B12 absorptions, we measured its plasma levels in two groups of HFD-fed rats before and after one-week gavage of B12 by 3 or 30 μM/kg/day. Our results indicated that in order to achieve the plasma B12 levels comparable to that in silymarin-treated rats, a ten times higher dose of dietary B12 administration (i.e., 30 μM/kg/day) is needed (Supplementary Fig. 5A). We then performed the silymarin and the higher-dose B12 administration experiments in germ-free C57BL/6 mice for 12 weeks. Our results demonstrated that the metabolic benefits on liver lipid metabolism by silymarin observed in obese rats disappeared in HFD-fed germ-free mice; in contrast, much lower liver TG and TC levels were observed in mice with direct B12 supplementation than the HFD control group (Fig. 5C, D); there were again no differences in body weight gain and food intake (Supplementary Fig. 5B). As expected, the plasma B12 in silymarin-treated mice is similar to that in the control group and much lower (by 50.3 pg/mL) than that in B12-treated mice (Fig. 5E). Pathological data on liver lipid droplets confirmed the protective roles of B12 against liver fat accumulation (Fig. 5F). Those results consolidated our finding that the action mechanisms of silymarin, at least to liver lipid metabolism, requires the gut microbiota and its B12 producing capabilities.

To explore the clinical importance of the B12-lipid links, we performed a retrospective study in 1030 inpatients with both serum B12 and triglycerides levels measured twice (the mean time interval is 11.8 weeks). Individuals within this cohort are generally elder (with mean age around 66.7 years) but have a normal range of BMI (23.4 kg/m^2^ in average) and balanced sex distribution (54.3% males) (Supplementary Data 4). We observed that there was a significant decrease of serum triglycerides (*P* = 2.31e−07; Wilcox signed-rank test) along the increase of B12 between the two time points (Supplementary Fig. 6A). Similar result was observed in 668 individuals with complete age, BMI and gender information but only 398 (59.6%) can be classified as responders to B12 (Supplementary Fig. 6B), suggesting that B12 supplementation may help lower serum triglycerides in some but not all individuals. Of interest, the baseline triglycerides of non-responders (mean value = 1.28 mmol/L) were much lower than that of the responders (mean value = 1.77 mmol/L) (*P* = 1.5e−15). It is thus possible that serum triglycerides are reluctant to change within a normal range similarly to the insignificant changes of fasting blood glucose upon metformin treatment in healthy individuals^27^, or alternatively those individuals may suffer from other factors that also regulate triglycerides such as folate^28^ and iron^29^. As expected, the delta changes of serum B12 associates negatively with the fluctuations of serum triglycerides (Supplementary Fig. 6C) and linear mixed-effect model revealed that 5.7% variations of serum triglycerides were explained by B12 levels in responders comparable to that explained by age and BMI (6.7%) (Fig. 5G).

To identify the potential gene target for microbially produced B12, we additionally performed liver transcriptome analyses in rats and germ-free mice supplemented with silymarin or B12 versus the HFD control groups. As expected, the one-carbon metabolism pathway interconnected with folate, B12, and the methionine cycles were significantly upregulated based on the liver transcriptome data in rats (Fig. 5H), demonstrating that the improvement of liver lipid metabolism upon silymarin or B12 treatment might act through this pathway and associated epigenetic regulation as previously proved in cell experiments^30^. In addition, we observed that the expression of key lipogenic genes or transcription factors such as *FASN*, *ACACB*, *SCD1*, and *SREBF1* were repressed whereas that of genes involved in fatty acid oxidation including *PPARA*, *CPT1*, and *BDH1* were increased by silymarin and B12; the expression levels of *FGFR4*^31^ and *GCK*^32^ known to enhance lipogenesis were also reduced (Fig. 5I). Of note, *MT1* and *MT2*, two genes encoding different metallothioneins which can protect against NAFLD via their antioxidant properties^33^, showed reduced expression levels in B12-treated rats. Further pathway enrichment analysis confirmed the upregulation of the fatty acid degradation pathway (Supplementary Fig. 6C).

**Fig. 6:**
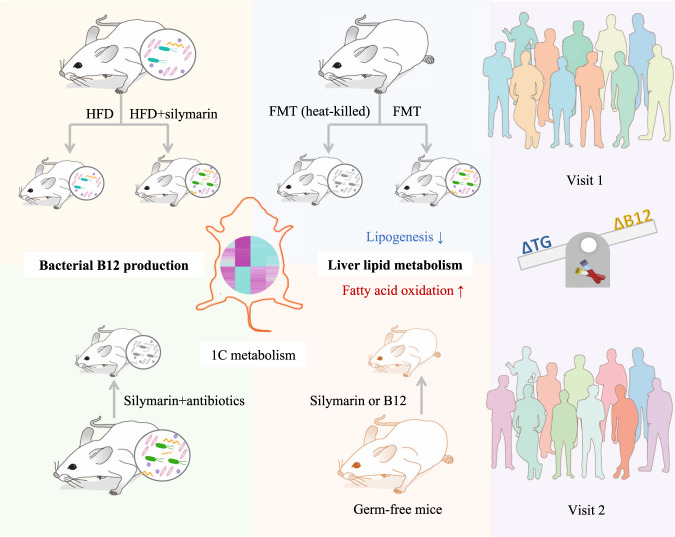
Main findings of the present study. Microbially produced B12 was shown to be responsible for the lipid-lowing effect of silymarin in both rats and germ-free mice. Additional liver transcriptome analyses demonstrated that the improvement in liver lipid metabolism was explained by reduced lipogensis and enhanced fatty acid oxidation. Moreover, the delta changes of serum B12 associated negatively with the fluctuations of serum triglycerides in humans.

In addition, liver transcriptome analyses in germ-free mice revealed that only 26 genes showed significant changes in silymarin-treated mice versus the HFD control group whereas 482 genes altered upon B12 treatment (Supplementary Fig. 5D and Supplementary Data 5). Targeted analyses showed that 9 out of 15 key markers genes for hepatic lipid metabolism were validated in germ-free mice upon B12 but not silymarin treatment (Fig. 5I), confirming that B12 potentially prevents liver fat accumulation by both suppressing lipogenesis and enhancing fatty acid oxidation. However, distinct with what observed in rats, no genes involved in one-carbon metabolism were found with significantly increased expression levels in B12-treated germ-free mice (Supplementary Data 5) and here the two genes encoding metallothioneins were surprisingly the top two increased genes by B12 administration (Fig. 5I and Supplementary Data 5). We reasoned that B12-mediated one-carbon metabolism and related epigenetic regulation of liver lipid metabolism require the gut microbiota and under gnotobiotic conditions, B12 might act indirectly via antioxidant and/or other uncharacterized pathways. Our findings were further summarized in Fig. 6.

## Discussion

Here we provided solid evidence showing that silymarin improved the liver lipid metabolism by altering the gut microbiota. The resulted gut microbial changes were associated with enrichment of bacterial B12 biosynthesis and increased fecal as well as plasma B12 concentrations. However, our results also implied that those alternations might be resulted from other components within silymarin rather than the traditionally considered silibinin through regulating gut redox potentials or other unknown mechanisms. Of note, silymarin-induced metabolic benefits on hepatic lipid metabolism were completely lost in germ-free mice, consolidating the suggestion that the action mechanism of this drug requires gut microbiota. Furthermore, transferring of the silymarin-adapted gut microbiota to rats fed on HFD showed alleviated triglycerides accumulation and increased expression of one-carbon metabolism pathway in the liver. Our retrospective study in humans provided additional evidence suggesting that the observed gut-liver crosstalk via B12 has potential clinical importance in regulating the lipid profile.

Growing evidence have been documented supporting that gut microbiota plays important roles in the pathogenesis and development of various metabolic diseases including obesity, NAFLD, and diabetes through interacting with the diet or directly with the host^34–36^. Several recent studies demonstrated that this new ‘metabolic organ’ could also affect the therapeutic effects of many oral non-antibiotic drugs^37,38^. Our findings here are well in line with those observations. For instance, many bacteria belong to *Blautia* (e.g., ASV24) and *Bilophila* (e.g., ASV37) which had been observed with increased abundance upon HFD feeding^14,16,39^ were confirmed in our study; increase of ASV5 (*A. muciniphila*) by silymarin/silibinin was consistent with previous studies intervened with dietary flavonoids or polyphenols^40,41^. However, despite the fact that silymarin could partially reverse some of those HFD-induced microbial changes, this drug in general shifted the gut microbiome towards a distinct direction than restoring it to a level resembling that of the controls. To illustrate, the HFD-induced increase of (*B. wadsworthia*) which is bile-tolerant^19^ was further aggravated by silymarin/silibinin treatment. One potential explanation might be that *B. wadsworthia* was sensitive to the environmental redox changes as the abundance of this bacterium was also found largely increased in vitro upon exposure of metformin^40^, another redox-modulating chemical. Similarly, abundance of *Escherichia*/*Shigella* was found increased by both silymarin and metformin^12^. Our FMT experiments provided direct evidence showing that the silymarin-adapted microbial changes could help improve liver lipid metabolism although not as dramatic as silymarin itself and in agreement, most variations of liver TG were explained by the gut microbiota.

Our pathway enrichment analyses revealed that silymarin treatment was associated with B12 biosynthesis and increased concentration of fecal as well as plasma B12. Low availability of B12 in the HFD and marked reduction of fecal B12 upon antibiotic treatment supported the idea that the increased fecal B12 by silymarin was largely due to bacterial biosynthesis. In support, silymarin-induced B12 increase disappeared in germ-free mice. Moreover, our gene screening analysis confirmed that B12 biosynthesis genes were very common in gut microbes including the aforementioned *B. wadsworthia*, *A. muciniphila*, and *Escherichia*/*Shigella*^42–44^. Experimental evidence had been recently provided to further support the B12-producing capability of *A. muciniphila*^45^. Interestingly, previous studies have demonstrated that the beneficial roles of this probiotic strain to glucose and lipid metabolism in the host were partially linked with a yet-to-be characterized membrane protein^46,47^. Currently, it is still unknown to what extent the B12-producing capability of this bacterium might also contribute to its probiotic potential. However, in this study, the variation explained by gut microbiota decreased from 82.4% to 27.5% after adjusting for fecal B12, indicating that the improved lipid metabolism by silymarin-adapted microbiota mainly attributed to bacterial B12 biosynthesis. Given the observations that silymarin possesses antioxidant properties^23^ and B12 biosynthesis is tightly regulated by environmental redox^24–26^, it is thus not surprising that supplementation of this drug could boost bacterial B12 production. In agreement, the concentrations of fecal B12 indeed correlated with the antioxidant capacity of the fecal samples. The parallel changes of fecal B12 but not folate upon treatment with this antioxidant suggested that biosynthesis of those two vitamins were distinctly regulated; it seems that bacterial production of folate depended more on the ratio between carbohydrate and protein in the diet as we previously shown^35^.

We further showed that B12 supplementation could help alleviate the HFD-induced liver fat accumulation in both rats and germ-free mice, well in line with the protective roles of dietary B12 against non-alcoholic steatohepatitis^48^. However, our results confirmed that the lipid profile impairment depended heavily on the circulating levels of B12. In support, previous mice experiments demonstrated that severe but not moderate B12 deficiency induced adiposity^49^. Additional liver transcriptome analyses suggested that silymarin and resulted bacterial B12 improved the liver lipid metabolism via suppressing lipogenesis and enhancing fatty acid oxidation. Importantly, the delta changes of plasma B12 negatively associated with the fluctuations of triglycerides in humans, pinpointing the clinical importance of dietary or bacterial B12 in dyslipidemia. Future studies should focus on silymarin-microbiota-B12 interactions in humans.

In conclusion, our results strongly support that the improvement of lipid metabolism by silymarin was tightly connected with regulation of gut microbiota and related bacterial B12 production. These findings are therefore of great importance for our understanding of the intricate interactions among diet, gut microbes, pathogenesis of NAFLD, and associated therapeutic options.

## Methods

### Animals

As female mice/rats resist to HFD-induced obesity and NAFLD, male mice/rats were used to induce obesity and NAFLD model by HFD^50^. Male Wistar (~180 g body weight; 8 weeks old) rats were purchased from Shandong Laboratory Animal Center with the permission number of SCXK 2014–0007 and raised under thermoneutral housing laboratory conditions (three-four per cage; a 12 h light/dark cycle and a temperature of 30–33 °C). Male C57BL/6J germ-free mice (*n* = 24) were purchased from and housed under germ-free conditions (four per cage; a 12 h light/dark cycle and a temperature of 22–24 °C) in Gempharmatech Co., Ltd. (Nanjing, China). Body weight, food intake and stool samples from all rats were measured/collected at the first and last weeks. All rats had free access to water and were fasted for 16 h before sacrifice (manual cervical dislocations or isoflurane inhalation) at the study end. The current animal study protocol was in accordance with international ethical guidelines and approved by the Animal Care and Use Committee of Institute of Biomedicine of Shandong University of Technology. All efforts were made to minimize animal suffering and reduce the number of animals used.

In the first experiment, 30 rats were divided into five groups (6 per group) and fed with NCD (4.2% crude fat; SYSE Bio-Tech. Co. Ltd., Jiangsu, China), HFD (D18040307; 45% of energy from fat from SYSE Bio-Tech. Co. Ltd., Jiangsu, China), HFD contains 0.1% silibinin, HFD contains 0.5% silibinin, or HFD contains 1% silymarin (which contains about 50% silibinin) for 12 weeks. Silymarin (purity > 98%) and silibinin (purity > 98%) were purchased from Nantong Feiyu Co. Ltd., Jiangsu, China.

Another rat experiment was performed to determine the therapeutic effect of vitamin B12 on liver lipid metabolism. 18 rats divided in three groups were fed with NCD, HFD, or HFD together with 3 μM/kg/day of B12 for 12 weeks. The dose of B12 was chosen based on the estimated fecal vitamin B12 increase upon silymarin treatment. To confirm B12 absorption in vivo, we gavaged two groups of rats (n = 6 per group) with two different doses of B12 by 3 and 30 μM/kg/day, respectively, for one week. The rats were fasted for 16 h before measurement of plasma B12 concentrations.

Five-six-week-old germ-free mice were randomly assigned to three groups, i.e., two cages per group, and fed with HFD, HFD plus 1% silymarin, and HFD plus B12 (~0.5 g/kg diet and 30–40 μM/kg/day per mouse), respectively, for 12 weeks. Body weight and food intake were measured each week. All mice had free access to water and were fasted for 16 h before sacrifice at the study end.

### Fecal microbiota transplantation

FMT experiment was performed according to an established protocol with slight modification^51^. Fecal samples from donor rats fed with HFD and 1% silymarin were collected in sterile conditions. The collected feces (100 mg) were pooled and resuspended in 1 ml of sterile saline. Obtained solution was vigorously mixed for 10 s and then centrifuged at 1000 × *g* for 5 min. The supernatant served as the fresh transplant material was prepared 10 min before each FMT to prevent substantial bacterial changes in vitro. Two groups of recipient rats (6 per group) fed on HFD were inoculated daily with heat-killed (control; heated to 121 °C for 20 min) and fresh transplant materials (100 μl/g for each rat), respectively, by oral gavage for 12 weeks.

We then measured the heat stability of silymarin at the aforementioned heating conditions based on high performance liquid chromatography (HPLC). HPLC analyses were performed using a Shimadzu LC20AT system equipped with a SPD-M20A diode array detector and C18 column (4.6 × 250 nm, 5 μm). The samples for HPLC analysis were eluted for 0–40 min with a gradient of 10–90% acetonitrile and 0.1% formic acid, for 40.01–50 min with 90% acetonitrile and 0.1% formic acid, and for 50.01–60 min with 10% acetonitrile and 0.1% formic acid at a flow rate of 1.0 ml/min. The detection wavelength was 280 nm.

### Antibiotics treatment experiment

We set up a 12-week experiment to explore whether antibiotics treatment could affect fecal concentrations of B12. A more therapeutic antibiotic exposure regime which could induce gut microbiota dysbiosis with lasting effects on the host was adopted here^52^. In particular, tylosin tartrate (obtained from Sigma Aldrich Co. Ltd., Saint Louis, USA) was dissolved in a sterile saline to achieve a dose of about 315 mg/kg/day. Similar to the FMT experiment, two groups of rats transplanted with either inactivated or fresh transplant materials for four weeks were exposed to tylosin tartrate for one week. Six more rats transplanted with fresh transplantation materials without antibiotic exposure was additionally included as the control group. After the antibiotic treatment, the rats were continued on the FMT experiment for another four weeks. Fecal samples were collected at weeks 4, 5, and 9 for measurement of B12.

### 16S rRNA and metagenomics profiling

Genomic DNA of each stool sample was extracted using E.Z.N.A.® soil DNA Kit (Omega Bio-tek Co. Ltd., Norcross, GA, USA) according to manufacturer’s protocols. For 16S rRNA sequencing, amplicon libraries covering the V3-V4 hypervariable regions were firstly amplified using primers 338F (5′-barcode-ACTCCTACGGGAGGCAGCA-3′) and 806R (5′-GGACTACHVGGGTWTCTAAT-3′)^53^. PCR reactions were performed in triplicate in 20 μL mixtures containing 4 μL of 5 × FastPfu Buffer, 2 μL of 2.5 mM dNTPs, 0.8 μL of each primer (5 μM), 0.4 μL of FastPfu Polymerase (Transgen Biotech Co. Ltd., Beijing, China), and 10 ng of template DNA with the following settings: 95 °C for 3 min, followed by 27 cycles at 95 °C for 30 s, 55 °C for 40 s, and 72 °C for 45 s and a final extension at 72 °C for 10 min. The resulted PCR products were extracted from a 2% agarose gel, purified using the AxyPrep DNA Gel Extraction Kit (Axygen Biosciences Co. Ltd., Union City, CA, USA), and further quantified using QuantiFluor™-ST (Promega Co. Ltd., USA). Purified and pooled amplicon libraries were paired-end sequenced (2 × 300) on the Illumina MiSeq platform (Illumina, San Diego, USA) according to the standard protocols by Majorbio Bio-PharmTechnology Co., Ltd. (Shanghai, China).

For metagenomics study, extracted DNA was fragmented to an average size of about 300 bp using Covaris M220 (Gene Company Limited, China) after examination of the DNA purity and quality with NanoDrop2000 and the 1% agarose gels electrophoresis system, respectively. The paired-end library was prepared by using TruSeqTM DNA Sample Prep Kit (Illumina, San Diego, CA, USA). Adapters containing the full complement of sequencing primer hybridization sites were ligated to the Blunt-end fragments. Paired-end sequencing was performed on Illumina HiSeq4000 platform (Illumina Inc., San Diego, CA, USA) at Majorbio Bio-Pharm Technology Co., Ltd. (Shanghai, China) using HiSeq 3000/4000 PE Cluster Kit and HiSeq 3000/4000 SBS Kits according to the manufacturer’s instructions.

16S reads were filtered, trimmed, merged, and analyzed using DADA2 pipeline (v1.20.0) with the amplicon error rates learned based on the built-in training models^54^. An ASV table was then constructed. Only reads with merged length between 400 and 432 bp were considered and all samples were rarefied to 16,439 reads before downstream analyses. Alpha and beta diversity analyses were calculated using phyloseq^55^. For the WGS datasets, after removal of the reads with low-quality and reads mapped to the rat genome, the resulted high-quality reads were mapped to the mice microbiome gene catalog and the largest human microbiome gene catalog with more than 15 million genes, respectively, using the MEDUSA pipeline (version 1). However, only the gene table resulted from the later catalog was used to produce the KEGG orthology profile since more reads were actually mapped to this gene catalog than to the mouse microbiome gene catalog^56^ and the largest human microbiome gene catalog with more than 15 million genes, respectively, using the MEDUSA pipeline^57^. However, only the gene table resulted from the later catalog was used to produce the KEGG orthology profile since more reads were actually mapped to this gene catalog than to the mouse microbiome gene catalog.

### Screening of bacterial B12 biosynthesis genes

55 query sequences representing over 40 key genes involved in bacterial B12 biosynthesis were blasted against 44,844,200 proteins from 12,551 bacteria with complete genome sequences (NCBI reference release 92). Note that 12 and 13 of those genes were involved in the aerobic and anaerobic pathways, respectively and an additional 15 were shared between those two B12 biosynthetic routes (Supplementary Data 3). The parameters used in the blastP searches were as following: e-value ≦ 1e−5; bit scores ≧ 40; alignment coverage for the query and subject sequences ≧50%.

### Liver RNA extraction, sequencing, and processing

Total RNA was extracted from fresh frozen liver tissues by using Trizol reagent (Invitrogen, USA).The quantity and quality of total RNA were determined by NanoDrop 2000 ultramicro-spectrophotometer (ThermoFisher Scientific, Waltham, MA, USA). Then, poly(A)-containing mRNA was enriched using magnetic beads with oligo(dT) and fragmented into small pieces using divalent cations under elevated temperature. The cleaved RNA fragments were reverse transcribed with Oligo T primer to produce cDNA, and dsDNA samples were generated under the action of DNA polymerase, RNase H enzyme, and T4 DNA ligase. Next, these cDNA fragments underwent processes including end repair, addition of a single ‘A’ base, and ligation with adapters. Finally, these products were purified and amplified through PCR to create the library for sequencing. Illumina HiSeq Xten/NovaSeq6000 was used for RNA sequencing.

Raw RNA-seq data were trimmed using Prinseq (v0.20.4)^58^ and then subjected to FastQC (v0.11.2)^59^ for read quality assessment. Trimmed reads were aligned to the *Rattus norvegicus* reference genome (release 6) or *Mus musculus* (release 10) from the UCSC genome browser database^60^ using the STAR aligner (v2.7.9a)^61^. The number of reads per gene was quantified by HTSeq (v0.9.1)^62^. Differentially expressed genes between groups were identified by R package DESeq2 (v1.32.0)^63^. KEGG pathway enrichment analyses using GOseq R package (v1.44.0)^64^ were performed after annotating each gene to the corresponding KEGG orthology based on KEGGREST package (v1.32.0)^65^.

### Biochemical and histological analyses

Fasting blood samples were centrifuged at 4000 × *g* for 5 min at 4 °C to obtain the plasma. The main tissues were immediately excised and stored in liquid nitrogen for biochemical analysis or in 10% formalin for histological studies. The levels of TC and TG in the plasma and liver were measured with commercially available kits (Jiancheng Co. Ltd., Nanjing, China). The concentrations of TNF-α and IL-6 in the plasma were determined using a commercial ELISA kit (Cusabio Co. Ltd., Wuhan, China) based on the manufacturer’s instructions. The fecal concentrations of six types of vitamins including vitamin A, B1, B2, B9, B12, and D3 were measured with commercially available kits (from Jiancheng Co. Ltd., Nanjing, China and Cusabio Co. Ltd., Wuhan, China). The total antioxidant capacity of collected fecal samples was also measured with commercially available kits (from Jiancheng Co. Ltd., Nanjing, China and Cusabio Co. Ltd., Wuhan, China). For histological analyses, tissue samples were fixed in formalin or 4% paraformaldehyde solution for 24 h and then embedded in paraffin. The liver sections (~3-μm thick) were stained with hematoxylin-eosin or oil red. The stained samples were observed at ×400 magnification. The liver lipids (% staining area) were analyzed by Image J (https://imagej.nih.gov/ij/).

### The clinical data

We searched the electronic health record database documented within the last five years in Ruijin hospital/Luwan branch, and screened 1030 inpatients with measurement of both fasting plasma TG and vitamin B12 at two different time points. Of those, 668 individuals also have available information on age, BMI, and gender. This retrospective analysis was approved by the Ethics Committee of Shanghai Ruijin Hospital Luwan Branch.

### Statistical analyses

All statistical analyses were done in the R environment (version 4.1.1)^66^. Wald test within the DESeq2 package (v1.32.0)^63^ was used to access the gut bacteria that were significantly changed between groups. Unless as otherwise indicated, nonparametric two-tailed Wilcox rank-sum test and spearman correlation were used for all animal experiments to determine the group differences and the relationships between different variables, respectively. Linear and random forest regression analyses were performed to calculate the variations of liver TG explained by fecal B12 and/or gut microbiota. The best random forest regression model was chosen with the minimum root-mean-square error calculated based on 5-fold cross-validation repeated 10 times using caret package^67^; ntree was set to 5000 and mtry was set to a range of values between 1 and 160. Wilcox signed-rank test was used to access the TG differences between two different measurements along the increase of plasma B12 in humans and linear mixed-effect model was then used to quantify the linear relationship between those two variables with the individual IDs as the random variable. Raw P values were adjusted by the Benjamini–Hochberg method^68^.

### Reporting summary

Further information on research design is available in the Nature Portfolio Reporting Summary linked to this article.

## Supplementary information

Supplementary Information

Description of Additional Supplementary Files

Supplementary Data 1

Supplementary Data 2

Supplementary Data 3

Supplementary Data 4

Supplementary Data 5

Reporting Summary

## Data Availability

Gut microbiome and liver transcriptome data were deposited at China NGDC Genome Sequence Archive (https://ngdc.cncb.ac.cn) with access numbers CRA008527 and CRA008528, respectively. Source data are provided with this paper.

## Source data

Source Data
