# Supplementary material for: Microbially produced vitamin B12 contributes to the lipid-lowering effect of silymarin
Source: Nat Commun. 2023 Jan 30;14:477. doi: 10.1038/s41467-023-36079-x (PMC9887073; doi:10.1038/s41467-023-36079-x)
Supplement: Supplementary file 1 — Supplementary Information [file 41467_2023_36079_MOESM1_ESM.pdf]

Supplementary Information

Microbially produced vitamin B12 contributes to the lipid-lowering effect of silymarin

Wen-Long Sun[1,6], Sha Hua[2,6], Xin-Yu Li[1], Liang Shen[1,*], Hao Wu[3,4,*], Hong-Fang Ji[1,5*]

[1]Institute of Biomedical Research, School of Life Sciences and Medicine, Shandong University of Technology, Zibo, Shandong, China

[2]Department of Cardiology, Ruijin Hospital/Luwan Branch, Shanghai Jiao Tong University School of Medicine, South Chongqing Rd. No.149, Shanghai, China.

[3]State Key Laboratory of Genetic Engineering, Human Phenome Institute, and Department of Bariatric and Metabolic Surgery, Huashan Hospital, Fudan University, Shanghai, China

[4]Zhangjiang Fudan International Innovation Center, Fudan University, Shanghai, China

[5]School of Life Sciences, Ludong University, Yantai, Shandong, China.

[6] These authors contributed equally.

Supplementary Figures 1-6 are deposited in the Supplementary Information.

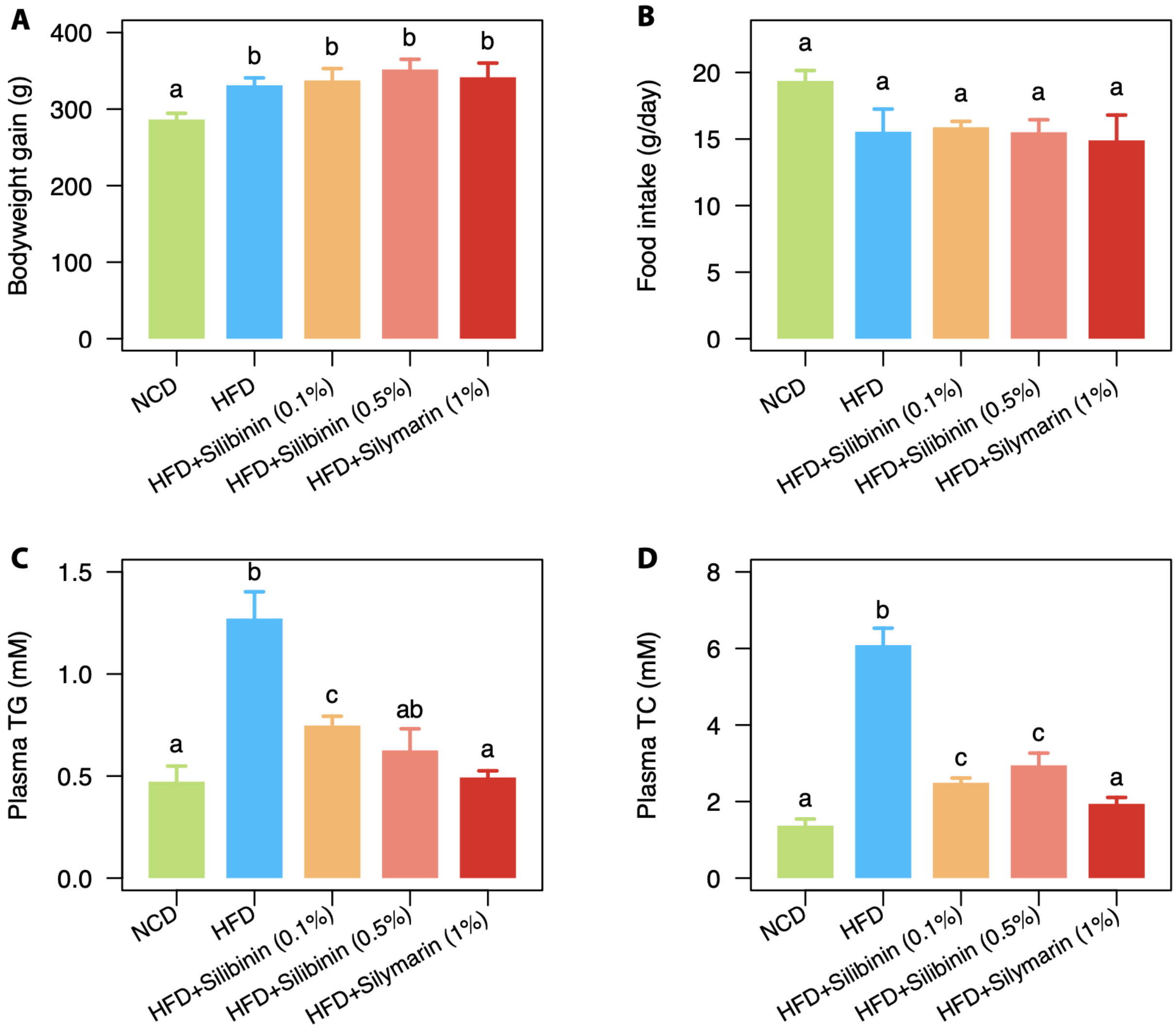

Supplementary Figure 1. Body weight gain, food intake, and plasma lipids in different treatment groups. Body weight gain (A), food intake (B), plasma TG (C) and plasma TC (D) in different treatment groups were estimated. Groups labeled with different letters indicate significant statistical differences (adjusted $P < 0.05$; Wilcox rank-sum test). NCD, normal chow diet; HFD, high fat diet; TG, triglycerides; TC, total cholesterol.

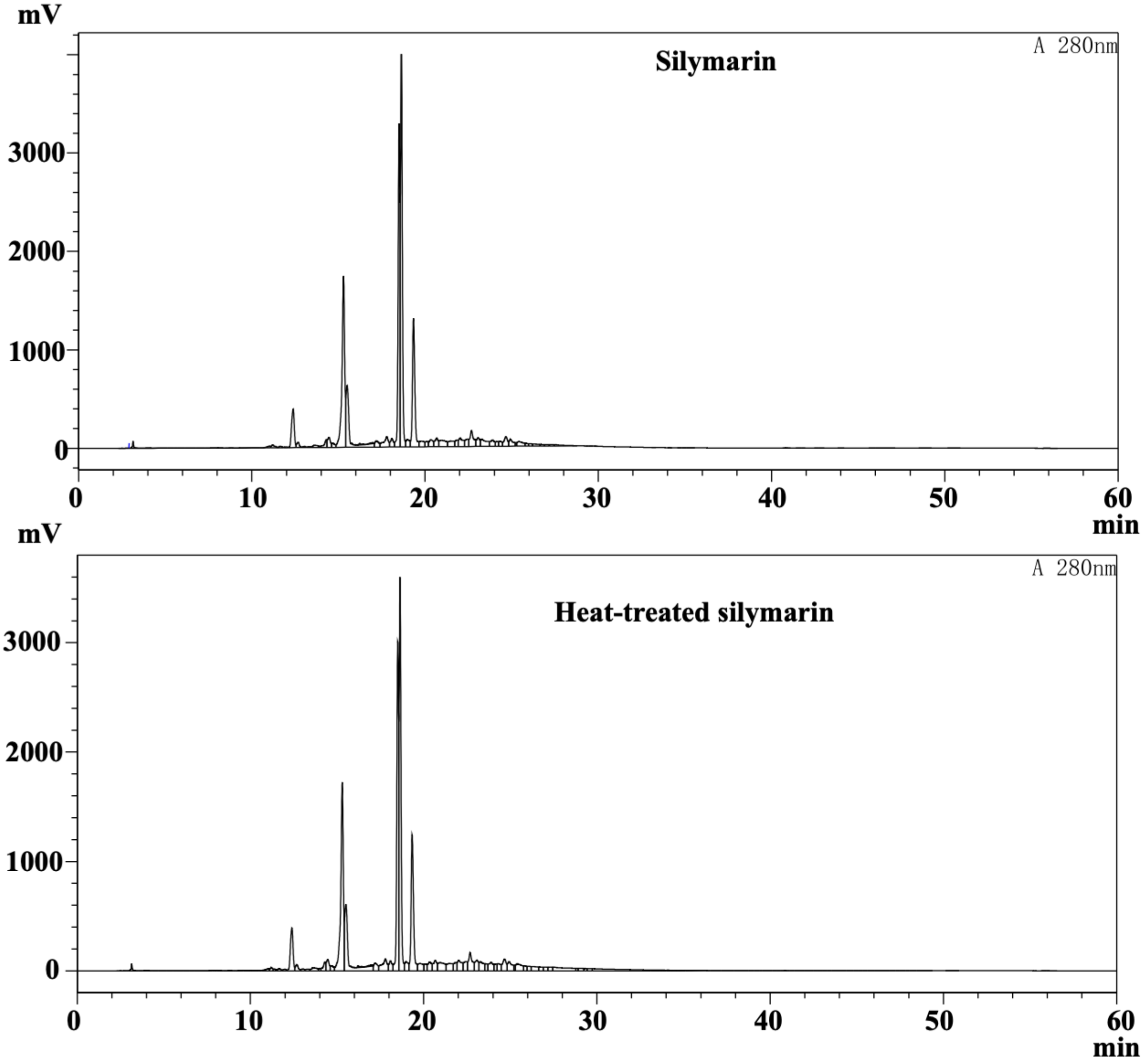

Supplementary Figure 2. Heat stability test of silymarin before and after the heat process by HPLC.

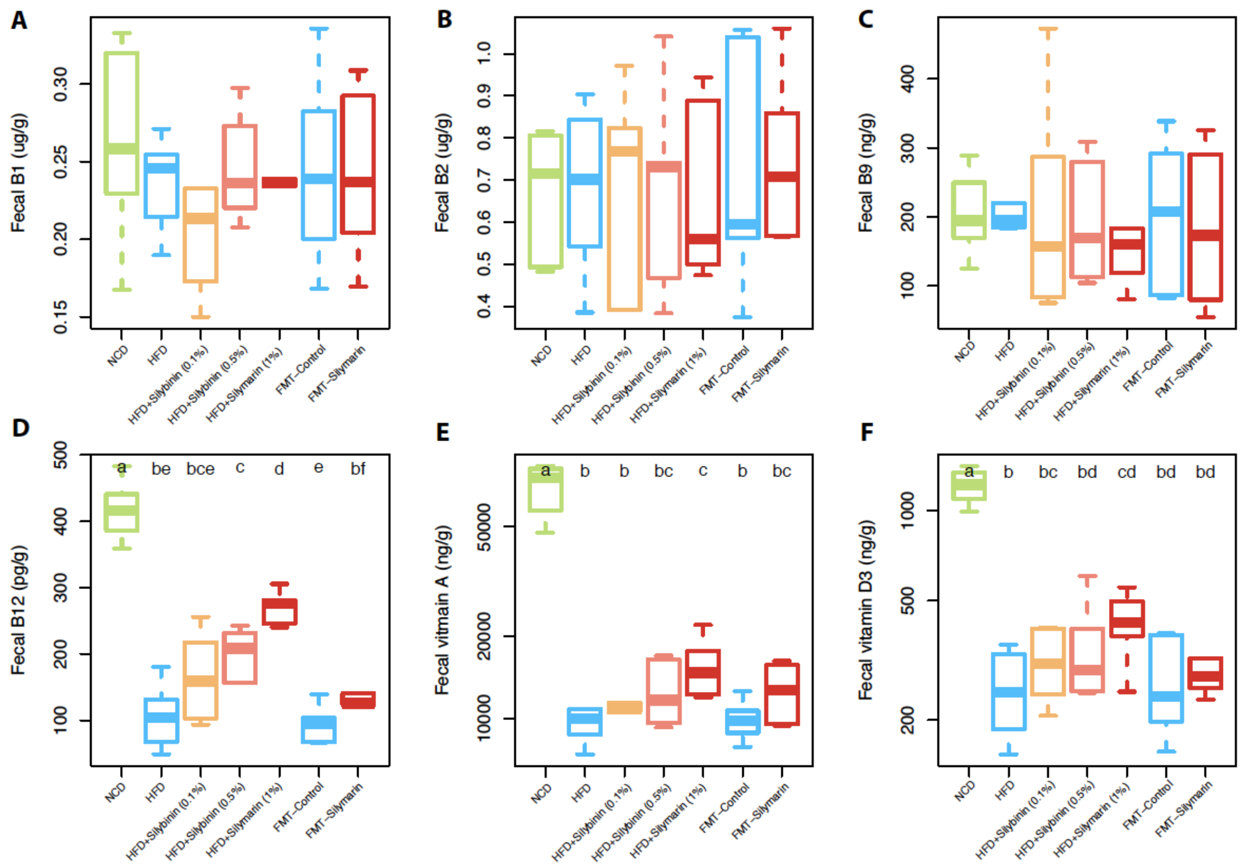

Supplementary Figure 3. Fecal concentrations of six vitamins in different treatment groups. No group differences were found for vitamins B1, B2, and B9 (panels A-C) and Groups labeled with different letters in panels D-F indicate significant statistical differences (adjusted P < 0.05; Wilcox rank-sum test). NCD, normal chow diet; HFD, high fat diet; FMT, fecal microbiota transplantation.

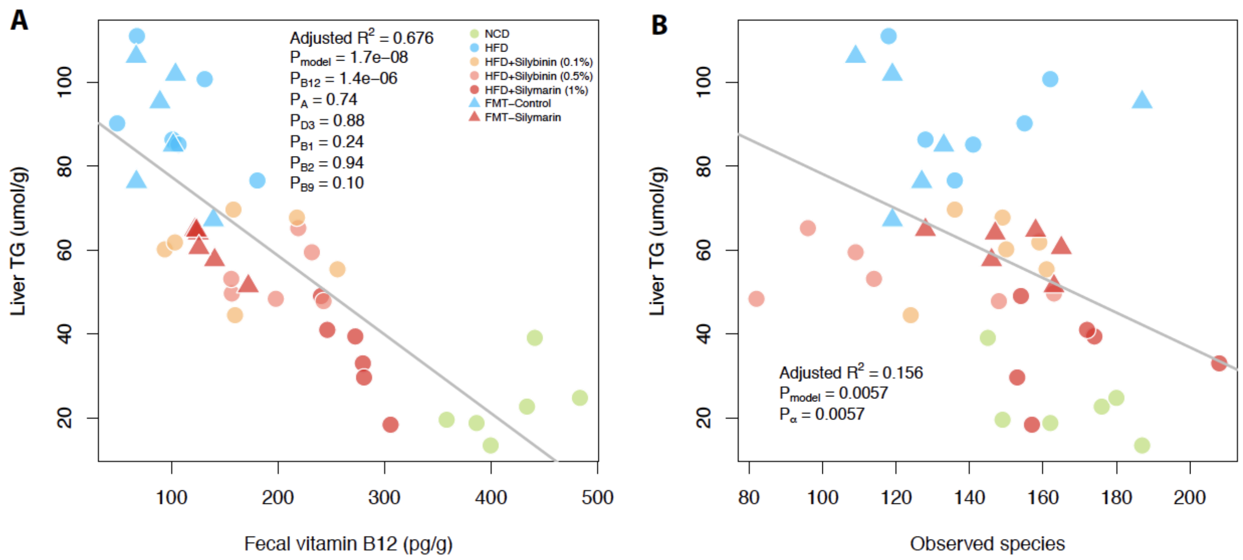

Supplementary Figure 4. Variations of liver TG explained by fecal vitamins (A) and bacterial alpha diversity (B) based on linear regression analyses. P value for each vitamin was also indicated in panel A. NCD, normal chow diet; HFD, high fat diet; TG, triglycerides; FMT, fecal microbiota transplantation.

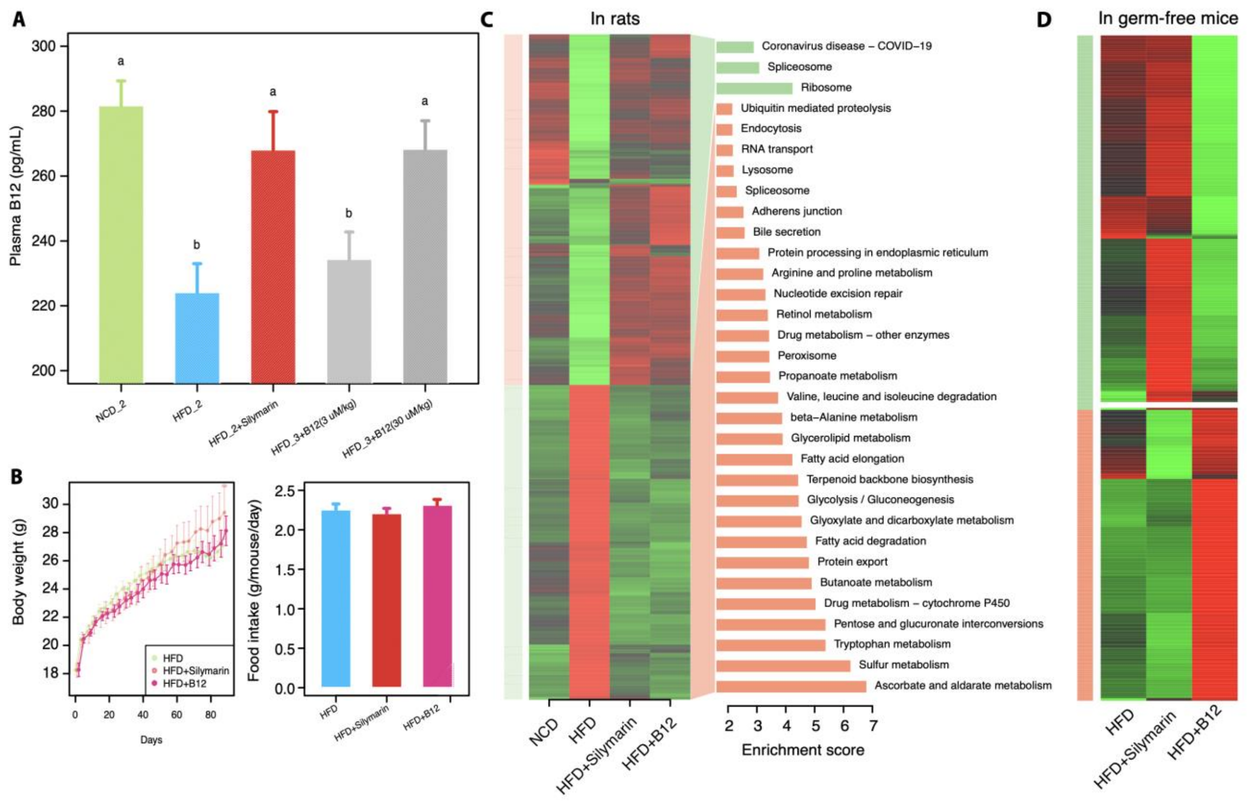

Supplementary Figure 5. Silymarin and B12 supplementation in rats and germ-free mice. (A) B12 absorption experiment using two different doses (for one week) as compared with three treatment groups; groups labeled with different letters indicate significant statistical differences (adjusted P < 0.05; Wilcox rank-sum test). (B) Body weight and food intake in three groups of germ-free mice. (C) Pathway enrichment analysis for rat liver transcriptome data. (D) Liver transcriptome data showing the expression levels of 26 genes changed by silymarin and 482 genes changed by B12 versus the HFD control group in germ-free mice (no KEGG pathway enrichment analysis was performed due to too few numbers of genes identified here).

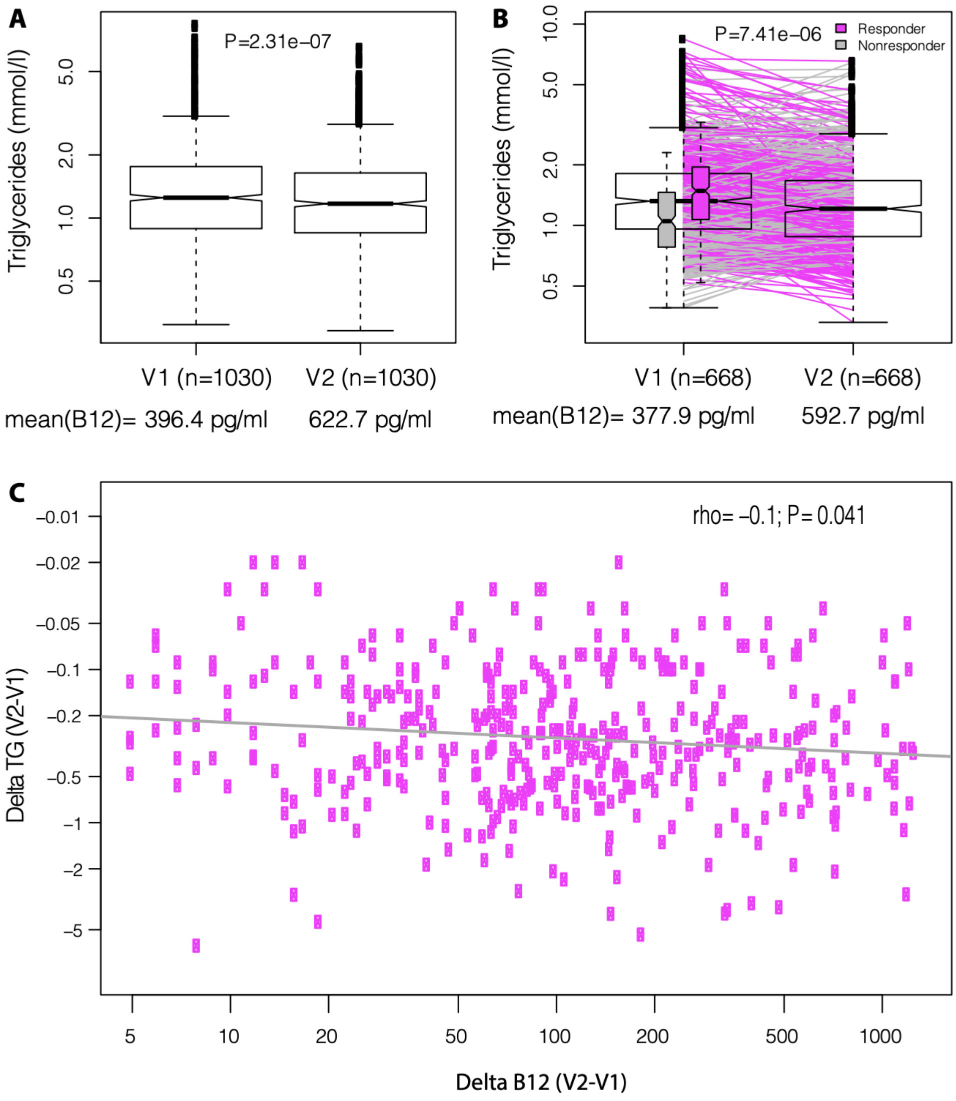

Supplementary Figure 6. Association between serum triglycerides and vitamin B12. (A) Levels of serum triglycerides along with increased serum vitamin B12 from visit (V1; mean value for B12 = 396.4 pg/ml) to visit 2 (V2; mean value for B12 = 622.7 pg/ml). (B) Levels of serum triglycerides in those individuals with complete information for age, BMI, and sex; changes of serum triglycerides along increased B12 were labeled by purple and grey colors, respectively. (C) linear relationship between delta changes of serum triglycerides and B12 in responders defined in (B).
