# Supplementary material for: Microbially produced vitamin B12 contributes to the lipid-lowering effect of silymarin
Source: Nat Commun. 2023 Jan 30;14:477. doi: 10.1038/s41467-023-36079-x (PMC9887073; doi:10.1038/s41467-023-36079-x)
Supplement: Supplementary file 2 — Description of Additional Supplementary Files [file 41467_2023_36079_MOESM2_ESM.pdf]

Description of Additional Supplementary Files

Supplementary Data 1. All significantly changed microbes across groups. The mean abundances for the top 100 abundant microbes were also shown.

Supplementary Data 2. The 110 KOs strongly associated with liver TG levels. Only KOs with spearman correlation coefficient larger than 0.9 or smaller than -0.9 were shown.

Supplementary Data 3. Genes involved in bacterial B12 biosynthesis. "Yes" indicates the existence of the query sequence in the corresponding bacterium. Genes involved in the aerobic and anaerobic B12 biosynthetic pathways were labeled with different background color. Several bacterial strains potentially affected by silymarin were also colored.

Supplementary Data 4. Basic clinical variables for the cohort study of B12 and serum triglycerides.

Supplementary Data 5. Hepatic genes altered by B12 administration in germ-free mice.
