# Supplementary material for: Microbially produced vitamin B12 contributes to the lipid-lowering effect of silymarin
Source: Nat Commun. 2023 Jan 30;14:477. doi: 10.1038/s41467-023-36079-x (PMC9887073; doi:10.1038/s41467-023-36079-x)
Supplement: Supplementary file 8 — Reporting Summary [file 41467_2023_36079_MOESM8_ESM.pdf]

nature portfolio

Corresponding author(s): Liang Shen, Hao Wu, Hong-Fang Ji

Last updated by author(s): Dec 22, 2022

Reporting Summary

Nature Portfolio wishes to improve the reproducibility of the work that we publish. This form provides structure for consistency and transparency in reporting. For further information on Nature Portfolio policies, see our Editorial Policies and the Editorial Policy Checklist.

Statistics

For all statistical analyses, confirm that the following items are present in the figure legend, table legend, main text, or Methods section.

n/a	Confirmed	
☐	☒	The exact sample size (n) for each experimental group/condition, given as a discrete number and unit of measurement
☐	☒	A statement on whether measurements were taken from distinct samples or whether the same sample was measured repeatedly
☐	☒	The statistical test(s) used AND whether they are one- or two-sided<br>*Only common tests should be described solely by name; describe more complex techniques in the Methods section.*
☐	☒	A description of all covariates tested
☐	☒	A description of any assumptions or corrections, such as tests of normality and adjustment for multiple comparisons
☐	☒	A full description of the statistical parameters including central tendency (e.g. means) or other basic estimates (e.g. regression coefficient) AND variation (e.g. standard deviation) or associated estimates of uncertainty (e.g. confidence intervals)
☐	☒	For null hypothesis testing, the test statistic (e.g. F, t, r) with confidence intervals, effect sizes, degrees of freedom and P value noted<br>*Give P values as exact values whenever suitable.*
☒	☐	For Bayesian analysis, information on the choice of priors and Markov chain Monte Carlo settings
☐	☒	For hierarchical and complex designs, identification of the appropriate level for tests and full reporting of outcomes
☐	☒	Estimates of effect sizes (e.g. Cohen's d, Pearson's r), indicating how they were calculated

Our web collection on statistics for biologists contains articles on many of the points above.

Software and code

Policy information about availability of computer code

Data collection	No software was used for data collection.
Data analysis	Open source softwares were used for data analysis throughout the manuscript. In brief, MEDUSA pipeline (version 1) was used for WGS and DADA2 (v1.20.0) for 16S metagenomics data analyses, respectively. Raw liver RNA sequencing data were preprocessed using Prinseq (v0.20.4) and FastQC (v0.11.2) and analyzed using the STAR (v2.7.9a)-HTSeq (v0.9.1) pipeline as stated in the manuscript. DESeq2 (v1.32.0), goseq (v1.44.0), and KEGGREST (v1.32.0), caret (v6.0-88) packages were used for statistical testing, pathway enrichment and regression analyses in the R environment (version 4.1.1).

For manuscripts utilizing custom algorithms or software that are central to the research but not yet described in published literature, software must be made available to editors and reviewers. We strongly encourage code deposition in a community repository (e.g. GitHub). See the Nature Portfolio guidelines for submitting code & software for further information.

Data

Policy information about availability of data

All manuscripts must include a data availability statement. This statement should provide the following information, where applicable:

- Accession codes, unique identifiers, or web links for publicly available datasets
- A description of any restrictions on data availability
- For clinical datasets or third party data, please ensure that the statement adheres to our policy

The Rattus norvegicus reference genome (release 6) or Mus musculus (release 10) used for transcriptome analysis were downloaded from the UCSC genome

browser database. Gut microbiome and liver transcriptome data were deposited at China NGDC Genome Sequence Archive with access numbers CRA008527 and CRA008528, respectively.

Human research participants

Policy information about studies involving human research participants and Sex and Gender in Research.

Reporting on sex and gender	Clinical data for both male and female subjects were collected, analyzed, and indicated in this study.
Population characteristics	Individuals within this cohort are generally elder (with mean age around 66.7 years) but have a normal range of BMI (23.4 kg/m2 in average) and balanced sex distribution (54.3% males); all those three variables were considered as covariates
Recruitment	This is a retrospective study in 1030 inpatients with both serum B12 and triglycerides levels measured twice according to their electronic health records.
Ethics oversight	The Ethics Committee of Shanghai Ruijin Hospital Luwan Branch

Note that full information on the approval of the study protocol must also be provided in the manuscript.

Field-specific reporting

Please select the one below that is the best fit for your research. If you are not sure, read the appropriate sections before making your selection.

[x] Life sciences [ ] Behavioural & social sciences [ ] Ecological, evolutionary & environmental sciences

For a reference copy of the document with all sections, see nature.com/documents/nr-reporting-summary-flat.pdf

Life sciences study design

All studies must disclose on these points even when the disclosure is negative.

Sample size	As other similar animal experiments, six to eight mice/rats were used per group.
Data exclusions	All collected data were used for analysis.
Replication	Improved lipid metabolism by silymarin were replicated in a second experiment and shown in Figure 1; silymarin-gut microbiota interactions were repeatedly shown by fecal microbiota transplantation experiments, antibiotics treatment experiment, and germ-free mice experiments; improved liver lipid metabolism by B12 supplementation were confirmed both in rats and in germ-free mice.
Randomization	All rats and mice were randomized to different treatment groups.
Blinding	The investigators were binded to group allocation during data collection but not during data analyses . However, data analyses were done by two bioinformaticians from a distinct city with no prior knowledge on silymarin.

Reporting for specific materials, systems and methods

We require information from authors about some types of materials, experimental systems and methods used in many studies. Here, indicate whether each material, system or method listed is relevant to your study. If you are not sure if a list item applies to your research, read the appropriate section before selecting a response.

Materials & experimental systems

n/a	Involved in the study	
x		Antibodies
x		Eukaryotic cell lines
x		Palaeontology and archaeology
	x	Animals and other organisms
	x	Clinical data
x		Dual use research of concern

Methods

n/a	Involved in the study	
x		ChIP-seq
x		Flow cytometry
x		MRI-based neuroimaging

Animals and other research organisms

Policy information about studies involving animals; ARRIVE guidelines recommended for reporting animal research, and Sex and Gender in Research

Laboratory animals	Male Wistar (~ 180 g body weight; 8 weeks old) rats were purchased from Shandong Laboratory Animal Center; C57 BL/6J germ-free mice (n=24;5-6 weeks old) were purchased from and housed (four per cage) in Gempharmatech Co., Ltd. (Nanjing, China).
Wild animals	The study did not involve wild animals.
Reporting on sex	Only male rats and mice were considered to exclude the potential confounding effect from sex.
Field-collected samples	Male Wistar (~ 180 g body weight; 8 weeks old) rats were raised under thermoneutral housing laboratory conditions (three-four per cage; a 12h light/dark cycle and a temperature of 30-33 °C). Male C57BL/6J germ-free mice (n=24) were housed under germ-free conditions (four per cage; a 12h light/dark cycle and a temperature of 22-24°C) in Gempharmatech Co., Ltd. (Nanjing, China)
Ethics oversight	The current animal study protocol was in accordance with international ethical guidelines and approved by the Animal Care and Use Committee of Institute of Biomedicine of Shandong University of Technology.

Note that full information on the approval of the study protocol must also be provided in the manuscript.

Clinical data

Policy information about clinical studies

All manuscripts should comply with the ICMJEguidelines for publication of clinical research and a completedCONSORT checklist must be included with all submissions.

Clinical trial registration	N/A
Study protocol	N/A
Data collection	N/A
Outcomes	N/A
